# Unveiling trends: insights into medicines reimbursement recommendations and health technology management in Ireland (2018–2023)

**DOI:** 10.1017/S0266462326103717

**Published:** 2026-04-06

**Authors:** David Aluga, Claire Gorry, Michael Barry, Paula Byrne, Paddy Gillespie, Susan M. Smith

**Affiliations:** 1 Discipline of Public Health and Primary Care, Institute of Population Health, School of Medicine, Trinity College Dublin, Ireland; 2 Health Service Executive-Medicines Management Programme, Trinity Centre for Health Sciences, St. James’s Hospital, Dublin, Ireland; 3 Department of Pharmacology and Therapeutics, School of Medicine, Trinity College Dublin, Dublin, Ireland; 4 Centre for Health Research Methodology, College of Medicine, Nursing and Health Sciences, University of Galway, Ireland; 5 Health Economics and Policy Analysis Centre, J.E. Cairnes School of Business & Economics, University of Galway, Ireland

**Keywords:** health technology assessment, reimbursement, orphan drugs, health technology management, managed access protocols

## Abstract

**Objectives:**

Health technology management (HTM) involves systems designed to ensure the safe, effective, and cost-effective use of health technologies following reimbursement. This study examines the recommendations of Ireland’s Health Service Executive-Drugs Group (HSE-DG), exploring the characteristics and patterns of positive or conditional positive recommendations and HTM-related requirements.

**Methods:**

We reviewed the minutes of HSE-DG meetings between January 2018 and December 2023. Data on medicines reviewed during this period were extracted into Microsoft Excel and analyzed narratively.

**Results:**

Over the study period, the HSE-DG reviewed 192 medicines (including new medicines and new indications for existing medicines), of which 157 received positive (115) or conditional positive recommendations (42). Of these, thirty-three were subject to HTM conditions, typically involving a managed access protocol or a specific reimbursement application process. Due to inconsistent reporting of key information, quantitative analysis was not feasible. However, common characteristics among HTM-linked recommendations emerged. These included submissions for reimbursement targeting a subset of the licensed population (45.5 percent vs. 5.6 percent in non-HTM cases), designation as orphan medicines (39.4 percent vs. 29.8 percent), and having both first-in-class and new chemical entity designations (75.8 percent vs. 47.6 percent).

**Conclusions:**

Findings indicate an increasing trend toward positive/conditional positive reimbursement recommendations with HTM in the Irish setting, with an average of 21 percent of positive/conditional positive recommendations over the study period contingent on HTM. More granular and consistent reporting of key indicators would enable the determination of characteristics associated with reimbursement recommendations with HTM.

## Key points for Decision-makers

- In Ireland, health technology management (HTM) is provided for under Section 20 (1) of the Health (Pricing and Supply of Medical Goods) Act of 2013, and there are different measures used to operationalize these provisions.
- From January 2018 to December 2023, the Health Service Executive-Drugs Group (HSE-DG) considered 192 medicines (including new medicines and new indications for existing medicines), out of which 157 received positive (115) and conditional positive (42) reimbursement recommendations. Thirty-three of these recommendations were subject to the establishment of HTM.
- Common characteristics among HTM-linked recommendations for reimbursement included submissions for reimbursement targeting a subset of the licensed population, designation as orphan medicines, and having both first-in-class and new chemical entity designations.

## Introduction

The decision to adopt or reimburse a new health technology may demand establishing systems to manage its use, so that the value of the health technology can be realized in practice. Health technology management (HTM) can be defined as the different measures put in place to enhance the safe, effective, and cost-effective use of health technologies post-reimbursement (1;2;3). Despite the existence of formal processes for the reimbursement of health technologies in European countries, the literature on the post-reimbursement use of HTM specifically is scarce (4). In Ireland, the Health Service Executive (HSE), which is responsible for implementing the government’s policies in providing health and social services in both the hospital and community settings, established the Medicines Management Programme (MMP) in 2013 to promote safe, effective, and cost-effective prescribing (5).

HTM in Ireland is implemented in line with Section 20 (1) of the Health (Pricing and Supply of Medical Goods) Act of 2013, which allows the Executive to apply conditions to the supply of an item on the reimbursement list (6). Section 20 (2) outlines the types of conditions that may be applied. Different approaches have been used to operationalize the provisions of Section 20. Early initiatives on existing medicines on the reimbursement list, not contingent on Section 20 of the Act, included the preferred drugs initiative, which encourages prescribers to make the preferred drug their first choice when prescribing a drug from a particular therapeutic class. This has evolved to HTM increasingly being applied from the outset of reimbursement through the use of managed access protocols (MAPs) – eligibility criteria for a medicine, based on licensed indication, cost-effectiveness, and with individual reimbursement applications required. Other mechanisms not contingent on Section 20 of the Act but put in place by the HSE to control expenditure on medicines include reference pricing and measures relating to generic availability and substitution.

The Health Act of 2013 also established the reimbursement list for medicines and non-medicinal products provided under the publicly funded Community Drug Schemes [6]. This Act empowers the HSE to add or remove an item on the list depending on its safety, clinical effectiveness, and cost-effectiveness, ensuring appropriate use of the item or appropriate allocation of health resources (6). To operate this function, the HSE-Drugs Group (HSE-DG) is charged with considering applications by marketing authorization holders (MAHs), submitted under the National Application, Assessment, and Decision Process for new medicines and new uses of existing medicines, and recommending the addition of medicines to the reimbursement list to the HSE leadership. This national committee consists of 15 members from various healthcare disciplines, health economics, and three public interest representatives, of which two are patient representatives and one is an ethicist (7;8). The HSE-DG meets approximately once every month to consider such applications with the HSE’s Corporate Pharmaceutical Unit (CPU) acting as its Secretariat. These interactions between the HSE and MAHs (under the umbrella of the Irish Pharmaceutical Healthcare Association [IPHA]) are further codified by the framework agreement on the supply and pricing of medicines (9).

**Figure 1.**
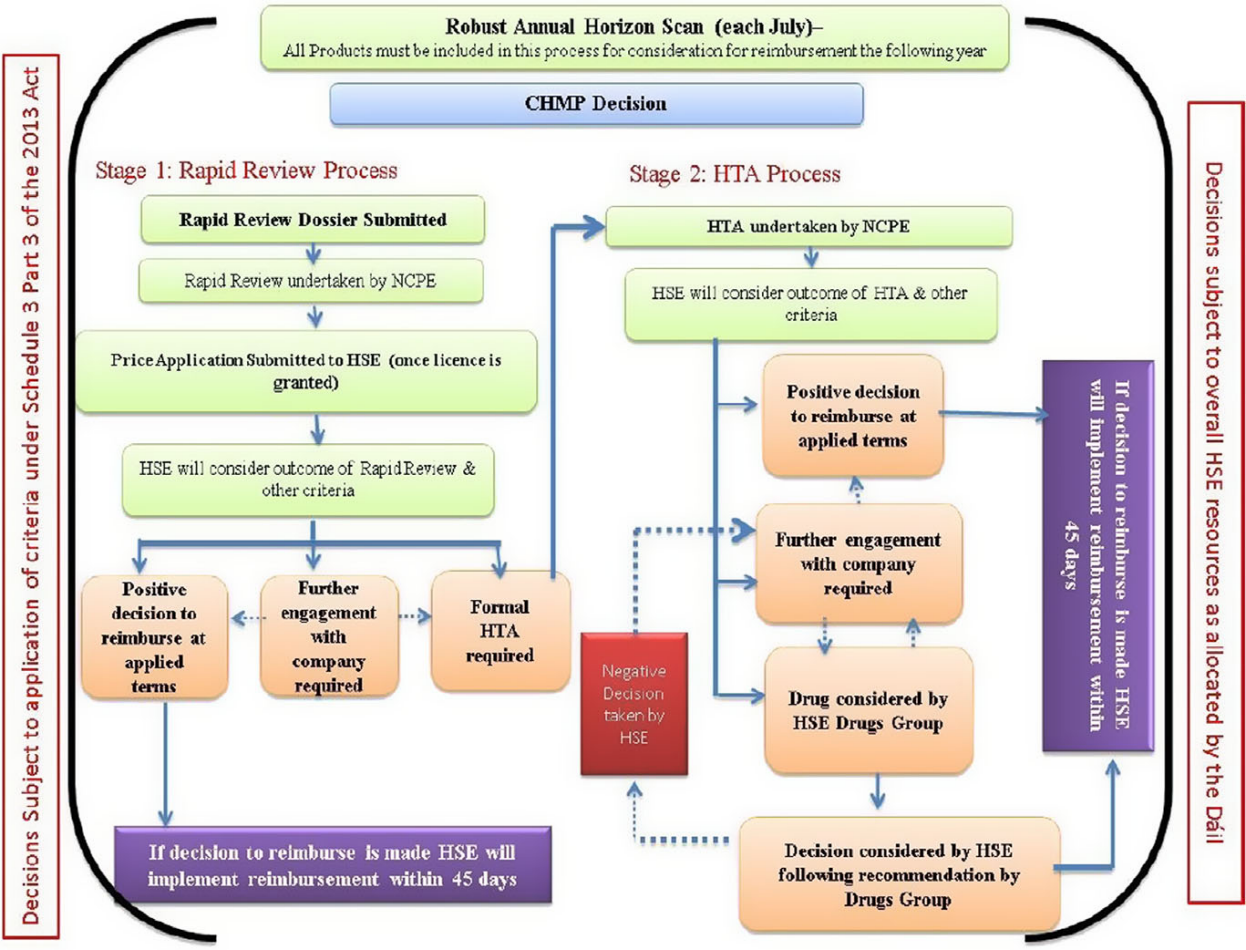
Diagram of the assessment process in Ireland. CHMP: European Medicines Agency’s Committee for Medicinal Products for Human Use (9).

MAHs who receive marketing authorization for their medicine from either the European Medicines Agency (EMA) or the Health Products Regulatory Authority (HPRA) in Ireland can apply through the CPU for the product to be added to the reimbursement list (10). On submission of an application, the CPU commissions the National Centre for Pharmacoeconomics (NCPE) to appraise the rapid review submitted by the applicant, a process taking ~4 weeks (11). The NCPE recommends to the CPU whether a full health technology assessment (HTA) is required to inform a reimbursement recommendation, or if a reimbursement recommendation can be made based on the information provided in the rapid review. A full HTA is not recommended when there is clear evidence that the medicine should or should not be reimbursed (12). The HSE-DG makes the recommendation to the HSE management regarding medicines reimbursement in line with the statutory criteria outlined in the Health Act Schedule 3, Part 3, which includes the clinical need for the item, the clinical and economic evidence, the budget impact, and the resources available to the Executive. The HSE applies the same process for assessment and decision-making for both medicines listed on the reimbursement list and hospital medicines, as shown in Figure 1 (9).

On considering the available evidence, the HSE-DG often gives one of three recommendations to the HSE: not to reimburse, to reimburse, or reimbursement subject to certain conditions. The common conditions for reimbursement are either an improved commercial offering by the MAH, the implementation of HTM, or both. Researchers have described the pharmacoeconomic evaluation process in Ireland (13). A recent report commissioned by the Department of Health found the process was producing results in line with international norms, is strongly evidence-based, and operating in line with the legislation; however, it was noted there was a lack of transparency regarding the number of applications being received and how these progress through the process (7). Studies have suggested factors affecting reimbursement decision-making, such as therapeutic classification, cost-effectiveness, budget impact estimates, safety and tolerability, and quality of evidence (14–18). This study aimed to examine the HSE-DG reimbursement recommendations to identify trends in recommendations for managed medicines reimbursement with HTM in Ireland and characterize the types of medicines for which HTM is recommended.

## Methods and materials

### Study design and setting

We reviewed materials resulting from decision-making processes by HSE-DG from January 2018 to December 2023 (19). This study period was chosen to maximize the number of whole years included and is limited by the public availability of the HSE-DG minutes of meetings. Information on all medicines considered by the group was reviewed, and key details about each reimbursement recommendation were extracted into a Microsoft Excel spreadsheet. DA extracted the data, which CG cross-checked to ensure its accuracy.

### Variables and data sources

The data extracted included the date of the meeting, names of the medicine, and MAH applying for the medicine to be added to the reimbursement list, population/indication, and reimbursement scheme for which it was considered, reimbursement, and HTM recommendations. Further information, such as the World Health Organization’s Anatomical Therapeutic Chemical (ATC) classification (20), cost per patient, gross projected budget impact, new chemical entity, first-in-class status, orphan drug or oncology designations, type of marketing authorization, and NCPE recommendation, were obtained from the Summary of Product Characteristics, technical summary, and similar reports using the search function on IPHA’s medicines.ie, European Medicines Agency (ema.europa.eu), National Centre for Pharmacoeconomics (ncpe.ie), and Health Products Regulatory Authority (hpra.ie) websites, where available.

Additionally, we reviewed common characteristics of medicines that received positive/conditional positive recommendations with HTM by the HSE-DG. These included factors such as therapeutic indication and area, MAH application for reimbursement in a subgroup of the licensed population, and the scheme under which it applied for reimbursement. We also considered the cost per patient per year or treatment course, the 5-year gross drug budget impact, and the NCPE recommendation following appraisal of the rapid review or HTA. A positive recommendation means that the HSE-DG recommends that the medicine be added to the reimbursement list, while a conditional positive recommendation means that the committee recommends that the medicine be added to the reimbursement list, generally subject to improved commercial offering by the MAH.

We classify a drug as a first-in-class medicine if it uses a new and unique mechanism of action to treat a disease (21), while a new chemical entity is a chemical compound that contains an active ingredient never before approved or marketed in any form (22). Orphan medicinal products are designed to diagnose, prevent, or treat life-threatening or severely debilitating conditions that impact no more than 5 out of every 10,000 individuals in the European Union (23). Orphan designation was assigned based on the designation by the EMA at the time of the medicine’s authorization for the indication in question.

### Reimbursement schemes

In Ireland, payments for public healthcare are made through various reimbursement schemes (24). High-tech medicines, dispensed through community pharmacies under the High Tech Drug Arrangements, are typically high-cost, hospital-prescribed medications (24). Other prescribed medicines fall under the Community Drug Schemes. They include various schemes like General Medical Services (GMS), Hospital Emergency, Long Term Illness (LTI), and Drugs Payment Scheme (DPS) (24).

### Data analysis

The extracted data were cleaned, deduplicated, and analyzed using Microsoft Excel for descriptive statistics on trends in positive/conditional positive recommendations. Recommendations were classed as negative or positive/conditional positive, and whether they involved any form of HTM, including the establishment of a Managed Access Protocol (MAP). Positive/conditional positive recommendations were grouped together for the purposes of this analysis, as medicines that received a conditional positive do not generally return to DG for confirmation of the positive decision. Positive/conditional positive recommendations were expressed as a percentage of the total recommendations made by the HSE-DG in a given year. The medicines were assigned to the year the final recommendation was made if they returned to HSE-DG multiple times.

It was not possible or appropriate to explore predictors for a positive/conditional positive reimbursement recommendation with HTM using a regression model due to the high prevalence of missing data on the characteristics of medicines considered by the HSE-DG. Specifically, there was incomplete information on whether the MAH applied for reimbursement in a subgroup of the licensed population, the reimbursement scheme under which it applied, and economic indicators such as cost per patient per year or treatment course and cumulative 5-year gross drug budget impact. Therefore, we conducted a narrative synthesis of the medicines that received positive or conditional positive recommendations with HTM, making use of the available records from the websites and databases (25). The medicines were grouped according to the organ or system of the body on which they act and their therapeutic properties. We further examined differences in the characteristics of medicines that received positive or conditional positive recommendations with HTM and those that received similar recommendations without HTM.

## Results

**Figure 2.**
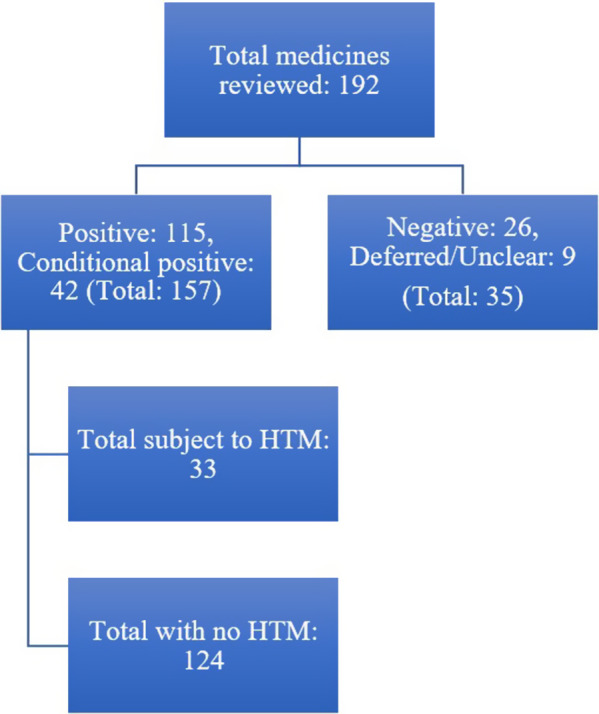
Flowchart of recommendations made by HSE-DG from 2018 to 2023.

The HSE-DG considered reimbursement recommendations for a total of 192 medicines (including new medicines and new indications for existing medicines) between January 2018 and December 2023 (Figure 2 and Table 1). Out of this number, the HSE-DG gave 157 (81.8 percent) positive or conditional positive reimbursement recommendations over the study period (*n* = 26, 23, 20, 33, 27, and 28 in 2018–2023, respectively). Of these, thirty-three medicines received a positive or conditional positive reimbursement recommendation with HTM (four in 2018, 2019, and 2020, eight in 2021 and 2022, and five in 2023).

**Table 1.** Outcome of applications by year and proportion of positive and conditional positive recommendations with HTM from 2018 to 2023

Year	Number of medicines considered	Number of positive and conditional positive	Number of positive and conditional positive with HTM	Percentage of positive and conditional positive with HTM
2018	31	26	4	15.4
2019	27	23	4	17.4
2020	23	20	4	20.0
2021	36	33	8	24.2
2022	37	27	8	29.6
2023	38	28	5	17.9
*Total*	*192*	*157*	*33*	*21.0*

While the number of medicines considered annually has trended upwards from thirty-one in 2018 to thirty-eight in 2023, the proportion of positive or conditional positive reimbursement recommendations contingent on HTM increased year-on-year between 2018 and 2022 before declining in 2023 (Table 1). In 2022, the highest proportion of reimbursement recommendations with HTM was seen at 29.6 percent, compared with 15.4 percent in 2018.

A summary of the characteristics of medicines recommended for reimbursement subject to HTM is provided in Table 2. Our findings showed that the thirty-three positive/conditional positive reimbursement recommendations with HTM by the HSE-DG belonged to ten different anatomical groups of the ATC classification system: alimentary tract and metabolism; blood and blood-forming organs; cardiovascular system; dermatologicals; anti-infectives for systemic use; antineoplastic and immunomodulating agents; musculoskeletal system; nervous system; sensory organs; and respiratory system. In addition, two therapeutic groups (Other drugs for disorders of the musculoskeletal system and Immunosuppressants) received three recommendations each, and only one therapeutic group (Other dermatological preparations) received four recommendations, which is the highest of any group. It is noteworthy that all four recommendations in the “Other dermatological preparations” therapeutic group were for the same drug (Dupilumab [Dupixent^®^]) used in one indication (atopic dermatitis) for three different subpopulations and another different indication (severe asthma). The remaining therapeutic groups received either one or two recommendations.

**Table 2.** Characteristics of positive/conditional positive reimbursement recommendations with HTM

Organ or system of action	Therapeutic group	Drug name	Therapeutic indication	First-in-class and/or new chemical entity	Orphan and/or oncology indication	Applied for reimbursement in subgroup of licensed population	Reimbursement scheme	Cost per patient per year or treatment course	Cumulative 5-year gross drug BI
Alimentary tract and metabolism	Other alimentary tract and metabolism products	Sapropterin	Phenylketonuria	Both	Orphan	No	High Tech Drug Arrangement	NR	€12.7 million
	Other alimentary tract and metabolism products	Sebelipase alfa	Lysosomal acid lipase deficiency	Both	Orphan	No	Hospital pricing approval	€1,460,066	€23.4 million
	Bile and liver therapy	Obeticholic acid (Ocaliva^®^)	Primary biliary cholangitis/cirrhosis	Both	Orphan	No	High Tech Drug Arrangement	€38,021.12	€21.5 million
	Drugs used in diabetes	Liraglutide (Saxenda^®^)	Obesity	Neither	No	Yes	Community Drug Schemes	€3,563.88	€10.2 million
	Bile and liver therapy	Cholic acid (Orphacol^®^)	Bile acid synthesis disorders	Both	Orphan	No	High Tech Drug Arrangement	NR	NR
Blood and blood-forming organs	Antithrombotic agents	Rivaroxaban	Coronary artery disease or peripheral artery disease	Neither	No	No	Community Drug Schemes	€962	€46.2 million
	Other hematological agents	Lanadelumab	Routine prevention of recurrent attacks of hereditary angioedema	Both	Orphan	Yes	High Tech Drug Arrangement	€236,056 to €471,368	€15.6 million
Cardiovascular system	Lipid modifying agents	Evolocumab (Repatha^®^)	Hypercholesterolemia	Both	No	Yes	High Tech Drug Arrangement	€8,046.33	€44.4 million
	Diuretics	Tolvaptan (Jinarc^®^)	Autosomal Dominant polycystic kidney disease	FIC	No	Yes	High Tech Drug Arrangement	€20,681.21	€17.8 million
	Cardiac therapy	Vericiguat (Verquvo^®^)	Long-term heart failure with reduced ejection fraction	Both	No	No	NR	€1,369.52	€5.7 million
	Lipid-modifying agents	Bempedoic Acid (Nilemdo^®^) and Bempedoic Acid in combination with Ezetimibe (Nustendi^®^)	Primary hypercholesterolemia (heterozygous familial or nonfamilial) or mixed dyslipidemia	Both	No	Yes	Community Drug Schemes	€705.51	€18.1 million
Dermatologicals	Other dermatological preparations	Dupilumab (Dupixent^®^)	Atopic dermatitis (adult)	Both	No	Yes	High Tech Drug Arrangement	€19,911	€51.9 million in the full population and €38.3 million in the “refractory” population.
	Other dermatological preparations	Dupilumab (Dupixent^®^)	Atopic dermatitis (adolescent)	FIC	No	Yes	High Tech Drug Arrangement	NR	NR
	Other dermatological preparations	Dupilumab (Dupixent^®^)	Atopic dermatitis (6–11years)	FIC	No	Yes	High Tech Drug Arrangement	NR	NR
	Other dermatological preparations	Dupilumab (Dupixent^®^)	Severe asthma (adults and adolescents 12 years and older)	FIC	No	Yes	High Tech Drug Arrangement	NR	NR
Anti-infectives for systemic use	Antivirals for systemic use	Letermovir (Prevymis^®^)	Prophylaxis of cytomegalovirus infection	Both	Orphan	No	High Tech Drug Arrangement	€19,315	NR
	Antivirals for systemic use	Fostemsavir (Rukobia^®^)	HIV–1 infection	Both	No	No	Hospital pricing approval	NR	NR
Antineoplastic and immunomodulating agents	Immunosuppressants	Tofacitinib	Ulcerative Colitis	Both	No	No	High Tech Drug Arrangement	NR	NR
	Immunosuppressants	Upadacitinib	Rheumatoid arthritis	Both	No	No	High Tech Drug Arrangement	NR	NR
	Immunosuppressants	Risankizumab (Skyrizi^®^)	Plaque Psoriasis	NCE	No	No	High Tech Drug Arrangement	€24,279	€43 million
	Antineoplastic agents	Larotrectinib (Vitrakvi^®^)	Solid tumors with a Neurotrophic Tyrosine Receptor Kinase gene fusion	Both	Oncology	No	High Tech Drug Arrangement	€163,552.20 per annum or €572,684 per treatment course.	€32 million
Musculoskeletal system	Other drugs for disorders of the musculoskeletal system	Nusinersen	Spinal muscular atrophy	Both	Orphan	Yes	Hospital pricing approval	NR	€38.2 million (both populations)
	Other drugs for disorders of the musculoskeletal system	Onasemnogene abeparvovec (Zolgensma^®^)	Spinal Muscular Atrophy	Both	Orphan	Yes	Bespoke arrangements	€1.75 million	€40.9 million
	Other drugs for disorders of the musculoskeletal system	Risdiplam	5q spinal muscular atrophy	Both	Orphan	No	High Tech Drug Arrangement	€264,371	€107 million
	Drugs for treatment of bone diseases	Romosozumab (Evenity^®^)	Osteoporosis	Both	No	Yes	High Tech Drug Arrangement	€8,640	€32.1 million
Nervous system	Psychoanaleptics	Idebenone (Raxone^®^)	Leber’s hereditary optic neuropathy	Both	Orphan	No	High Tech Drug Arrangement	€77,796	€3.7million
	Analgesics	Erenumab	Migraine	Both	No	Yes	High Tech Drug Arrangement	€7,804.06	€27.3 million
	Analgesics	Fremanezumab	Migraine prophylaxis	Both	No	Yes	High Tech Drug Arrangement	€8,919.59 and €8,671.48 (225mg once every month or 675mg once every three months respectively).	€49.6 million
	Other nervous system drugs	Patisiran	hereditary Transthyretin-mediated amyloidosis	Both	Orphan	No	Hospital pricing approval	€414,419.20	€59.2 million
	Other nervous system drugs	Tafamidis	Wide-type or hereditary transthyretin amyloidosis in adult patients with cardiomyopathy	Both	Orphan	No	High Tech Drug Arrangement	€140,401	€99.8 million
	Antiepileptics	Delta–9-tetrahydrocannabinol/Cannabidiol (Sativex^®^)	Multiple sclerosis-related spasticity	FIC	No	No	High Tech Drug Arrangement	€5,181	€6.2 million
Sensory organs	Ophthalmologicals	Voretigene neparvovec (Luxturna^®^)	Inherited retinal dystrophy caused by confirmed biallelic RPE65 mutations	Both	Orphan	No	Hospital pricing approval	€810,750	€7.2 million
Respiratory system	Drugs for obstructive airway diseases	Tezepelumab (Tezspire^®^)	Severe asthma (in adults and adolescents 12 years and older).	Both	No	Yes	High Tech Drug Arrangement	NR	NR

BI, budget impact; CE, cost-effectiveness; HTA, health technology assessment; FIC, first in class; NCE; new chemical entity; NR, not reported.

Almost half (fifteen out of thirty-three, 45.5 percent) of all the recommendations applied for reimbursement in a subgroup of the licensed population. The majority of the reimbursement applications recommended with HTM were for medicines reimbursed under the High Tech Drug Arrangement (twenty-three out of thirty-three, 70 percent) and then Hospital Pricing Approval (five out of thirty-three, 15 percent). There were significant variations in reported budget impact estimates among the recommendations, with cost per patient per year or treatment course ranging from €705.51 to €1.75 million and the cumulative 5-year gross drug budget impact ranging from €3.7 million to €107 million. The reimbursement application that had the highest cost per patient per year or treatment course (Onasemnogene abeparvovec [Zolgensma^®^]) was assessed through the Beneluxa Initiative. Furthermore, twenty-five (75.8 percent) recommendations were both first-in-class and new chemical entities, five (15.2 percent) recommendations were only first-in-class medicines, and one (3 percent) was a new chemical entity. Another thirteen (39.4 percent) were orphan medicinal products, and one (3 percent) was for an oncology indication.

It was not possible to explore the determinants for positive/conditional positive reimbursement recommendations with HTM statistically due to the widespread underreporting of key indicators. However, the characteristics of 124 positive or conditional positive reimbursement recommendations issued without HTM are summarized in Table 3. Notably, antineoplastic therapeutic groups (antineoplastic agents, antineoplastic agents and antibody–drug conjugates, and other antineoplastic agents) were overrepresented, comprising 66.2 percent of all reimbursement recommendations made without HTM. Our study also identified distinct patterns in the characteristics of medicines that received positive or conditional positive recommendations with HTM compared with those recommended without HTM. These included applications for reimbursement in a subpopulation of the licensed population; of applications for medicines with a positive/conditional positive reimbursement status and a recommendation for HTM, 45.5 percent of these were for a subpopulation of the licensed indication. By comparison, of the positive/conditional positive recommendations without HTM, only 5.6 percent of these were for a subpopulation of the licensed indication. Similarly, 39.4 percent of positive/conditional positive recommendations with HTM were for orphan medicinal products compared with 29.8 percent of those that received positive/conditional positive recommendations without HTM. Furthermore, 75.8 percent of positive/conditional positive recommendations with HTM were for medicines with both first-in-class and new chemical entity designations compared to 47.6 percent of positive/conditional positive recommendations without HTM. We also observed that the proportion of positive/conditional positive reimbursement recommendations with HTM was higher for medicines from specific therapeutic groups when compared to positive/conditional positive reimbursement recommendations without HTM: Other drugs for disorders of the musculoskeletal system (9.1 percent vs. 0.8 percent); Immunosuppressants (9.1 percent vs. 4.0 percent); and Other dermatological preparations (12.1 percent vs. 0 percent).

**Table 3.** Characteristics of positive/conditional positive reimbursement recommendations without HTM

Therapeutic group	Number of drugs	Percentage of total no. of drugs (%)	FIC	NCE	Both FIC and NCE	Orphan indication	Oncology indication	Applied for reimbursement in subgroup
All other therapeutic products	2	1.6	2	1	1	0	0	0
Anti-anemic preparations	1	0.8	1	1	1	0	0	0
Antibacterial for systemic use	4	3.2	4	3	3	1	0	0
Anti-epileptics	5	4.0	5	4	4	3	0	0
Antimycotics for systemic use	1	0.8	0	0	0	1	0	0
Antineoplastic agents	74	59.7	55	32	29	20	74	1
Antineoplastic agents and antibody drug conjugates	1	0.8	1	1	1	0	1	0
Anti-Parkinson drugs	1	0.8	0	0	0	0	0	0
Antithrombotic agents	1	0.8	1	1	1	0	0	0
Antiviral for systemic use	1	0.8	1	1	1	1	0	0
Drugs for obstructive airway diseases	2	1.6	0	2	0	0	0	0
Drugs for the treatment of bone diseases	1	0.8	1	1	1	1	0	1
Drugs used in diabetes	2	1.6	0	0	0	0	0	2
Endocrine therapy	2	1.6	1	1	1	0	2	0
Immunosuppressants	5	4.0	3	3	2	1	0	1
Other alimentary tract and metabolism products	4	3.2	3	3	3	3	0	1
Other antidepressants	1	0.8	1	1	1	0	0	0
Other antineoplastic agents	7	5.7	7	4	4	2	7	0
Other drugs for disorders of the musculoskeletal system	1	0.8	1	1	1	0	0	0
Other nervous system drugs	1	0.8	1	1	1	1	0	0
Other respiratory system products	2	1.6	2	1	1	2	0	1
Other therapeutic radiopharmaceuticals	1	0.8	1	1	1	1	1	0
Psycholeptics	1	0.8	1	1	1	0	0	0
Selective immunosuppressants	1	0.8	1	1	1	0	0	0
Urologicals	1	0.8	0	0	0	0	0	0
N/A	1	0.8	0	0	0	0	0	0
Total	124	100	93	65	59	37	85	7
Percentage (%)	100		75.0	52.4	47.6	29.8	68.5	5.6

FIC, first in class; N/A, not available; NCE, new chemical entity.

## Discussion

The objective of this research was to identify trends in recommendations for managed medicines reimbursement with HTM in Ireland and characterize the types of medicines for which HTM is recommended. We found that 157 out of the 192 medicines considered by the HSE-DG from 2018 to 2023 received either a positive or a conditional positive reimbursement recommendation. This number represented 81.8 percent of all the medicines for various indications considered by the HSE-DG during that period. Moreover, 33 (or 21 percent) of the 157 positive/conditional reimbursement recommendations were contingent on the establishment of HTM. The percentage of positive/conditional positive reimbursement recommendations contingent on HTM rose year-on-year from 2018 to 2022 before falling in 2023. This seems to follow a similar pattern to total budgetary allocations to health, which saw a steep rise in healthcare spending, exacerbated by the COVID-19 pandemic (26). Certain attributes tend to reoccur among medicines that received positive or conditional positive recommendations subject to HTM. These included applying for reimbursement in a subgroup of the licensed population, orphan status, having both first-in-class and new chemical entity designations, and seeking reimbursement under either the High Tech Drug Arrangement or Hospital Pricing Approval. Others are medicines belonging to specific therapeutic groups (Other drugs for disorders of the musculoskeletal system, Immunosuppressants, and Other dermatological preparations) and having significant budget impact estimates.

These findings support a previous study by Gorry et al. (14) on the development of MAPs to facilitate drug reimbursement in Ireland, which noted that MAPs were recommended when there is a high estimated budget impact, concerns about the possibility of off-label use, and uncertainties in clinical or cost-effectiveness, leading to reimbursement approval for a defined cohort of the full licensed population. Similarly, Schmitz et al. (15) conducted a retrospective analysis of reimbursement recommendations in Ireland and found that the incremental cost-effectiveness ratio and quality of evidence were the most important drivers of reimbursement recommendations. Other less crucial factors are the year in which the assessment was conducted, the level of uncertainty, and the safety and tolerability of the medicine (15). The growth in the use of reimbursement recommendations with HTM to manage cost-effectiveness and appropriate use of medicines may reflect a response by health care decision-makers to address some of the uncertainties in the cost-effectiveness and clinical evidence, as highlighted in Schmitz et al. (15). Over time, the form of HTM to be employed has become more prescriptive, with the adoption of the MAP seen from 2021 onward.

Regression results on factors influencing the rapid review outcome in Ireland showed a greater probability of HTA being recommended with certain therapeutic areas (endocrine, musculoskeletal, and neoplasm), first-in-class and orphan disease, and increasing annual drug costs per patient (16). However, these results have been criticized as a simplification of the complex factors associated with rapid review outcomes (17). A study by Varley et al. (18) found a positive and significant association between anticancer drugs, drugs designated first-in-class, and high-cost drugs with the likelihood of requiring a full HTA. They found no significant association for orphan drugs when indicators related to cost and clinical evidence were included in the model. This aligns with our findings that belonging to specific therapeutic groups, having high cost, and both first-in-class and new chemical entity designations were commonly occurring characteristics of medicines that received positive or conditional positive recommendations with HTM.

It is important to note that there are several medicines that have come through the pricing and reimbursement process during the period of this study that did not go to HSE-DG but are subject to HTM in the form of a MAP (27). These medicines were likely reimbursed on a cost-minimization basis without the need for consideration by HSE-DG (e.g., galcanezumab, eptinezumab, abrocitinib, tralokinumab, and alirocumab) (27). In addition, anticancer medicines more often require HTA and, therefore, are more likely to go to the HSE-DG, but to date have largely been excluded from these explicit HTM processes post-reimbursement. This explains the high proportion of antineoplastic therapeutic groups among medicines that received positive or conditional positive reimbursement recommendations without HTM. As of the end of 2025, 43 medicines (or specific uses of medicines) are reimbursed subject to MAPs operated by the MMP (27). Other medicines (like treatments for rheumatological conditions) are subject to other types of HTM, such as the implementation of validations regarding prescriber speciality, line of treatment, dosing, and treatment intervals, in the reimbursement claims system. Many of these medicines would not have been captured by our analysis in the absence of specific consideration by the HSE-DG, since many of these medicines do not require full HTA. As such, this study cannot be considered an exhaustive review of all HTM measures in place in Ireland but rather focused on one element of HTM – primarily MAPs.

A strength of this study was the use of real-world data on the reimbursement decision processes. However, restrictions on the study period due to the non-availability of HSE-DG minutes and incomplete data as a result of inconsistent reporting hindered the ability to conduct a robust statistical analysis to arrive at definite conclusions. Confidential pricing information is unavailable in public versions of the minutes, which renders interpretation of cost and budgetary information challenging. Furthermore, data on incremental cost effectiveness ratios for the medicines employed in the decision-making process could not be considered in the analysis. Finally, not every drug approved for reimbursement by the HSE goes to HSE-DG (due to the rapid review process), so information in the public sphere is incomplete. Our results suggest that an improved and consistent reporting of key indicators pertaining to pricing, budget impact, and characteristics of medicines from marketing authorization by EMA or HPRA until final approval for reimbursement by the HSE would enable clearer determination of characteristics of medicines recommended for reimbursement subject to HTM. While the findings of this study relate to the Irish decision-making context specifically, they will help other jurisdictions that formally employ HTA and HTM to identify some key parameters influencing the reimbursement decision-making processes, thereby enabling a more in-depth exploration of the subject. Other jurisdictions will also have additional or alternative context-specific variables that will need to be taken into consideration.

## Conclusion

This study found a rising trend in the proportion of positive/conditional positive reimbursement recommendations for medicines in Ireland from 2018 to 2022, before falling sharply in 2023. Potential determinants of reimbursement with HTM were application for reimbursement in certain therapeutic groups and subgroups of the licensed population, orphan status, having both first-in-class and new chemical entity designations, under High Tech Arrangement/Hospital Pricing Approval, and substantial budget impact estimates. There is a need for more detailed and consistent reporting of key indicators to enable determination of characteristics associated with reimbursement recommendations with HTM.

## Data Availability

The dataset may be shared upon reasonable request to the corresponding author.

## References

[1] Hegarty F, Amoore J, Blackett P, McCarthy J, Scott R. Healthcare Technology Management - A Systematic Approach. 1st ed. Florida: Taylor & Francis Group; 2017.

[2] Smith A, Barry M. Combining health technology assessment and health technology management to deliver cost-effective prescribing and cost containment - the Irish experience. Expert Rev Pharmacoecon Outcomes Res. 2020;20(5):431–436. 32909850 10.1080/14737167.2020.1822739

[3] T. Holman Medical device maintenance: A comprehensive approach to service management. Ohio, United States: GE Healthcare 2023 [cited 2025 May 08]. Available from: https://advantushp.com/wp-content/uploads/2024/03/GEHC_AdvantusHealthPartners_HTM_Whitepaper_branded_final-1.pdf

[4] le Polain M, Franken M, Koopmanschap M, Cleemput I. Drug reimbursement systems: international comparison and policy recommendations. Health Services Research (HSR). Brussels: Belgian Health Care Knowledge Centre (KCE); 2010 KCE Reports 147C. D/2010/10.273/90.

[5] Medicines Management Programme. The Medicines Management Programme aims to promote safe, effective and cost effective prescribing. [cited 2025 May 08]. Available from: https://www.hse.ie/eng/about/who/cspd/medicines-management/

[6] Government of Ireland Health (Pricing and Supply of Medical Goods) Act. S. 18, Houses of the Oireachtas. 2013. [cited 2025 May 08]. Available from: https://www.irishstatutebook.ie/pdf/2013/en.act.2013.0014.pdf

[7] Mazars. Review of the Governance Arrangements and the Resources currently in place to support the Health Service Executive reimbursement and pricing decision-making process. Dublin, Ireland: Department of Health. 2020 [cited 2025 May 08]. Available from: https://assets.gov.ie/258773/70942321-d13b-49b4-b24d-28e097076ba5.pdf

[8] Public Patient Involvement Hub. HSE drugs group. Dublin, Ireland: Irish Platform for Patients Organisations. 2021 [cited 2025 May 08]. Available from: https://ppihub.ipposi.ie/item/hse-drugs-group-2/

[9] Irish Pharmaceutical Healthcare Association. Framework agreement on the supply and pricing of medicines. 2021 [cited 2025 May 08]. Available from: https://www.ipha.ie/wp-content/uploads/2021/12/20211206_FASPM_TermsofAgreement_Final_ForSigning.pdf

[10] Health Service Executive. Corporate pharmaceutical unit. 2024 [cited 2025 May 08]. Available from: https://www.hse.ie/eng/about/who/cpu/

[11] National Centre for Pharmacoeconomics, Ireland. Overview of the drug reimbursement process. 2024 [cited 2025 May 08]. Available from: https://www.ncpe.ie/submission-process/overview-of-the-drug-reimbursement-process/

[12] National Centre for Pharmacoeconomics. NCPE recommendations. n.d. [cited 2025 December 08]. Available from: https://www.ncpe.ie/ncpe-recommendations/

[13] McCullagh L, Barry M. The Pharmacoeconomic Evaluation Process in Ireland. PharmacoEconomics. 2016;34(12):1267–1276. 27473640 10.1007/s40273-016-0437-5

[14] Gorry C, Daly M, Barrett R, et al. Utilising health technology assessment to develop managed access protocols to facilitate drug reimbursement in Ireland. Appl Health Econ Health Policy. 2024;22:771–781. 39133443 10.1007/s40258-024-00904-1 PMC11470914

[15] Schmitz S, McCullagh L, Adams R, Barry M, Walsh C. Identifying and revealing the importance of decision-making criteria for health technology assessment: A retrospective analysis of reimbursement recommendations in Ireland. PharmacoEconomics. 2016;34(9):925–937. 27034245 10.1007/s40273-016-0406-z

[16] Murphy A, Redmond S. To HTA or not to HTA: Identifying the factors influencing the rapid review outcome in Ireland. Value Health. 2019;22(4):385–390. 30975388 10.1016/j.jval.2018.10.011

[17] O’Donnell H, Lamrock F, Tilson L, Barry M. To HTA or not to HTA: Identifying the factors influencing the rapid review outcome in Ireland. Value Health. 2020;23(2):274–275. 32113633 10.1016/j.jval.2019.06.013

[18] Varley Á, Tilson L, Fogarty E, McCullagh L, Barry M. The utility of a rapid review evaluation process to a national HTA agency. PharmacoEconomics. 2022;40(2):203–214. 34635994 10.1007/s40273-021-01093-8 PMC8795027

[19] Health Service Executive. Drugs group minutes [cited 2025 May 08]. Available from: https://www.hse.ie/eng/about/who/cpu/drugs-group-minutes/

[20] World Health Organisation’s Collaborating Center for Drug Statistics Methodology. *Guidelines for ATC classification and DDD assignment 2024. Oslo, Norway.* 2023.

[21] Gu J, Wu Q, Zhang Q, You Q, Wang L. A decade of approved first-in-class small molecule orphan drugs: Achievements, challenges and perspectives. Eur J Med Chem. 2022;243:114742. 36155354 10.1016/j.ejmech.2022.114742

[22] Food and Drug Administration. New Chemical Entity Exclusivity Determinations for Certain Fixed-Combination Drug: Guidance for Industry. Maryland, United States: U.S. Department of Health and Human Services; 2014 [cited 2025 May 08]. Available from: https://www.fda.gov/files/drugs/published/New-Chemical-Entity-Exclusivity-Determinations-for-Certain-Fixed-Combination-Drug-Products.pdf?utm

[23] Directorate-General for Health and Food Safety. Orphan medicinal products: European Commission [cited 2025 May 08]. Available from: https://health.ec.europa.eu/medicinal-products/orphan-medicinal-products_en

[24] Flanagan S. Statistical analysis of claims and payments 2023. 2024 [cited 2025 May 08]. Available from: https://www.hse.ie/eng/staff/pcrs/pcrs-publications/hse-annual-report-2023.pdf

[25] Popay J, Roberts H, Sowden A, et al. *Guidance on the conduct of narrative synthesis in systematic reviews: A product from the ESRC Methods Programme*; 2006. 10.13140/2.1.1018.4643.

[26] Parliamentary Budget Office. Health spending in Ireland 2015 - 2023: Houses of the Oireachtas. 2023 [cited 2025 May 08]. Available from: https://data.oireachtas.ie/ie/oireachtas/parliamentaryBudgetOffice/2023/2023-08-15_health-spending-in-ireland-2015-2023_en.pdf

[27] Medicines Management Programme. Managed access protocols. [cited 2025 May 08]. Available from: https://www.hse.ie/eng/about/who/cspd/medicines-management/managed-access-protocols/

